# Editorial: Exploring HIV disclosure challenges and approaches around the globe

**DOI:** 10.3389/fpubh.2023.1357915

**Published:** 2024-01-09

**Authors:** Grace Gachanja, Gary J. Burkholder, Aimee Ferraro, Diego Ripamonti

**Affiliations:** ^1^School of Health Sciences, Walden University, Minneapolis, MN, United States; ^2^School of Psychology, College of Psychology and Community Services, Walden University, Minneapolis, MN, United States; ^3^School of Health Sciences, College of Health Sciences and Public Policy, Walden University, Minneapolis, MN, United States; ^4^Infectious Diseases Unit, ASST Papa Giovanni XXIII, Bergamo, Italy

**Keywords:** HIV disclosure, HIV-related stigma, perinatal HIV infection, HIV/AIDS, HIV quality of life

Despite dramatic improvements in HIV pharmacology over the past three decades, HIV-related stigma remains a major and persistent barrier to disclosure (1). Five years ago, *Disclosure Within HIV-Affected Families* confirmed that disclosure is a stressful and complicated process and offered interventions and recommendations to ease the process (2).

Advances in antiretroviral (ARV) treatment have led to undetectable plasma HIV RNA levels below 200 copies/ml, without onward sexual transmission, potentially contributing to stigma reduction (3). However, these advances have not reduced the burden of disclosure for people living with HIV (PLWH) to their families, friends, and sexual partners, and within school, work, and healthcare settings (3). Such barriers undermine healthcare access, adherence to medical care, socialization, family planning, and participation in HIV research (4, 5).

This second Research Topic expanded the scope of HIV disclosure research beyond the immediate family, globally, and across all time periods post-diagnosis, aiming to discover new HIV disclosure models and updated recommendations since previous research. Seven manuscripts, primarily original research, are featured, highlighting HIV stigma as a major obstacle to HIV disclosure in various settings and situations. This emphasizes the urgent need for interventions addressing HIV stigma to support those who disclose an HIV diagnosis. The manuscripts are briefly outlined below.

## Original research

Wanjala et al. reported on the experience of HIV-related stigma regarding ARV treatment in a rural Kenyan setting among 40 PLWH (aged 18–60 years). The findings confirmed the persistence of different forms of HIV-related stigma, leading to internalized stigma, non-disclosure, anxiety, depression, isolation, poorer mental health, and diminished sexual or marital prospects for unmarried PLWH, which impacted ARV adherence and care-seeking behavior. The authors advocated for the urgent implementation of anti-stigma programs to improve the quality of life for PLWH.

Moraa et al. reported on the HIV disclosure experiences of 28 youth living with HIV (YLH) aged 14–19 years and 24 caregivers of YLH in schools in Kenya. Disclosure to school staff was an important step to attending clinic visits and receiving support to store and adhere to medication while at school. Despite a fear of post-disclosure stigma, both YLH and caregivers acknowledged the benefits of disclosure to school staff. Disclosure to school staff was mostly positive, and confidentiality was upheld. The authors recommended school-based HIV disclosure processes that provide guidance to YLH and caregivers on whom to disclose and from whom to seek support.

Magill et al. reported results from a randomized study of 146 non-disclosing caregiver-child dyads that addressed factors associated with caregiver compliance with an HIV disclosure intervention in Kenya. Previous isolation of caregivers due to stigma significantly reduced disclosure, and children on longer ARV treatment were less likely to have their HIV status disclosed. The authors advocated for disclosure interventions directed toward improving mental health and ARV treatment adherence counseling, in addition to active engagement of newly prescribed children.

Aurpibul et al. explored the ongoing challenges of 20 young adults (median age 25 years) living with perinatal HIV (YAPHIV) in predominantly HIV-prevalent rural communities in Thailand. The majority of the participants received disclosure as children and described it as a neutral, non-emotional experience. However, YAPHIV entering adulthood faced challenges in disclosing HIV to friends and sexual partners due to fear of stigma and discrimination. The authors recommended that YAPHIV receive support with coping skills and disclosure efficacy to help them transition smoothly into adulthood.

Mugo et al. conducted a cross-sectional analysis of HIV disclosure involving 375 adolescents and young adults living with HIV (AYLH) in Kenya. The authors reported that most AYLH were perinatally infected, preferred disclosure by their caregiver before the age of 12, and had high levels of satisfaction with the pre- and post-disclosure support they received from their caregivers and healthcare workers (HCWs). The authors recommended that AYLH receive well-timed disclosure with support from caregivers and HCWs throughout the process.

## Brief research report

Bingaman et al. reported on the experiences of 24 parents of internationally adopted children with perinatally acquired HIV (IACP) living in the United States. The adoptive parents expressed fear of stigma, but their knowledge of HIV disclosure laws empowered them to support and guide the IACP through the disclosure process by identifying people who should be informed while balancing the need for privacy with advocacy for the IACP. The authors recommended community-wide stigma reduction programs and supportive training for IACP families during the disclosure process.

## Opinion

Cruz et al. used Goffman's theory to discuss issues facing adolescents and young adults living with HIV (YPHIV) in Brazil. They described YPHIV's experiences of HIV disclosure and stigma that hindered their medical care and socialization in the community. The authors argued that groups formed by stigmatized people, such as YPHIV, work together as a social support network to effectively reduce stigma and thereby assist YPHIV with a long-term illness who are transitioning into adulthood. The authors advocated for society to collaborate to tackle the challenges of living with HIV, rather than leaving the responsibility solely to the affected community.

## Conclusions

The United Nations Program on HIV/AIDS (UNAIDS) 90–90–90 targets monitoring the clinical health of PLWH. In 2016, a “fourth 90” target was proposed to improve the health-related quality of life (HrQoL) of PLWH by addressing comorbidities and psychosocial challenges (6). This second Research Topic shows that while more is understood about the HIV disclosure process, it remains challenging for all involved and is hampered by persistently high levels of HIV stigma.

A need for support for infected children and youth transitioning into young adulthood also emerged from the published research, revealing an unmet need for this expanding group. If we are to achieve the success of the UNAIDS “fourth 90” target, acknowledging the persistence of HIV stigma and integrating mental health services (7) are some of the first steps aimed at reducing negative perceptions of PLWH and improving HrQoL.

We encourage HCWs, school staff, social activists, political institutions, and social media influencers to work together in formulating educational processes that lead to community-based structured HIV stigma reduction interventions and programs that support HIV disclosure. Tackling stigma globally will favor virological control of HIV at the community level, improve HIV prevention strategies, and help PLWH live healthy lives.

## Author contributions

GG: Conceptualization, Writing – original draft, Writing – review & editing. GB: Conceptualization, Writing – original draft, Writing – review & editing. AF: Conceptualization, Writing – original draft, Writing – review & editing. DR: Conceptualization, Writing – original draft, Writing – review & editing.
